# Comparative analysis of China’s Health Code, Australia’s COVIDSafe and New Zealand’s COVID Tracer Surveillance Apps: a new corona of public health governmentality?

**DOI:** 10.1177/1329878X20968277

**Published:** 2021-02

**Authors:** Fan Yang, Luke Heemsbergen, Robbie Fordyce

**Affiliations:** Deakin University, Australia; Monash University, Australia

**Keywords:** citizenship, COVID-19, COVIDSafe, COVID Tracer, Health Code, platforms, privacy, public health surveillance

## Abstract

The onset of the COVID-19 pandemic and the subsequent lockdown of cities worldwide generated a dramatic increase in the use of public health trac(k)ing technologies. This article presents an empirical analysis of China’s Health Code on WeChat and Alipay, Australia’s COVIDSafe and New Zealand’s COVID Tracer. We ask: how does app-based public health monitoring differ from prior forms of state tracking and corporate surveillance, and interface with public and private ideals of health and citizenship? Based on a comparative analysis of the selected apps and the political economy that surrounds their code and implementation, we argue that there is a new corona of surveillance to address COVID-19 crises by intensifying the diffusion of national surveillance technologies and framing these into justifiable moral practice. In conclusion, we identify a new ‘corona’ of public health governmentality during COVID-19 pandemic through an intensification of top-down institutional data extraction from human bodies.

## Introduction

This entry in the *Extraordinary Issue* responds to the first year of the COVID-19 pandemic through engagement with state management of Coronavirus Disease as a public health problem, as if there were an app for that. Differing app-based public health management techniques have led to new forms of surveillance of civilians, as well as new justifications. Governments were quick to use non-pharmaceutical interventions (NPIs) in response to the COVID-19 pandemic as a way to slow down the spread of the disease, given the absence of a vaccine and adequate distribution networks. A historical retrospect of governance around epidemics and pandemics in the last century, including the 1918 pandemic caused by H1N1 and other notable viruses from the H2N2 virus in 1957 to 1958, the H3N2 in 1968 and the 2009 H1N1 flu pandemic (Tognotti, 2013), suggests that nation-states had reason to extensively embed NPIs of social distancing and contact tracing during the 2020 COVID-19 pandemic into digital platforms. Epidemiological patterns from 2019 suggested that COVID-19 transmission strongly correlates with how populations congregate and socialise. To intervene in the chain of infections, states employed specific technologies and different governmental rationalities guided by technological solutionism and economic centralism to produce unique responses within each national context. We argue that assuming an ‘IT-mediated gaze’ (French, 2014) into everyday, material practices of health surveillance exposes the mythology of data truth and the complex socio-technological platforms that manage what is made visible. At the same time, smartphones and Bluetooth called into question the *meaning* of public health surveillance – with traditional distinctions between surveillance of disease from surveillance of individuals (French and Monahan, 2020) becoming less clear.

To remind the reader of the extraordinary nature of the time: organised institutional responses to COVID-19 asked residents to begin staying at home to ‘save lives’ and ‘flatten the curve’. But the pandemic was also framed in unprecedented dire economic terms, as manifest in a hitherto unseen spike of unemployment, recession and fears of further outbreaks collapsing economic activity (which had been ‘paused’) all together (Farr, 2020). In light of this, a variety of app-based surveillance technologies were used by states to frame a recovery of both economy and public health through solutions that stopped the spread and allowed individual quarantines to be lifted, and thus return to robust and ‘healthy’ economic activity (Goggin, 2020). In contrast to the extraordinary COVID-19 public health needs, more ordinary app-based public health ‘surveillance’ initiatives included ideas around epidemiological data gathering by survey response (Navin et al., 2017) and various health promotion programmes (Lee et al., 2018).

Yet, COVID-19 digital surveillance solutions also departed from previous corporate and state-based solutions. These new systems differ from the familiar corporate capitalised surveillance conducted by Facebook or Google where convenience coincides with increasingly powerful and comprehensive data collection (Lyon, 2001; Zuboff, 2019). They are also different to forms of state tracking that have generally relied on static CCTV or court-authorised warrants to access discrete or nebulous data for later analysis. The large-scale and unpredictable duration of pandemic surveillance also distinguishes itself from the short-term and geolocational-confined disease surveillance conducted by public health officials in preplanned mass gatherings (Lombardo et al., 2008). The novelty of this new national form of surveillance technology leveraged active consent from citizens with mobile phones to form citizen-bodies-device networks that emanate what we term a *corona* of public health surveillance.

We deploy the term ‘corona’ to a data corona radiating above the health of human bodies in ‘appearance recalling the solar corona’ (Almeida et al., 1968) as subjects move about their everyday surveilled life. This solar corona description comes directly from how Almeida et al. (1968) described the appearance via electron microscopy of the virus type they so named.^1^ This form of data surveillance is situated just above the subject and expels data into (public) space through augmented proximity detection of citizens’ unique digital devices. Digital COVID tracking is imposed on citizens’ personal smart devices to synthesise their digital and physical footprints in the name of contact tracing. Intervening in the movements of individuals and surveilling their coronal proximity to others have been deemed crucial for pandemic surveillance in the People’s Republic of China (PRC), Australia and Aotearoa/New Zealand (NZ) for domestic disease control and economic recovery throughout the first months of the pandemic. These three countries have presented unique apps for mobile phones as solutions for this opportunity of coronal surveillance.

In our assessment on PRC’s Health Code, Australia’s COVIDSafe app and NZ’s COVID Tracer app, we argue that these surveillance technologies vary substantially in functionalities, governance models and operations, but their vision collectively gestures towards individualising the responsibility of public health in the interest of economic recovery. This novel action and justification of public health does not displace other existing forms of surveillance but integrates aspects of corporate, state and individual surveillance that show how the works of Gillespie (2010) and French (2014) combine in digital governance responses to pandemics. This mix has yielded new public anxiety and resistance on individual privacy protection grounds, which is surprising compared to more invasive, yet standardised forms of digital surveillance integral to capitalism and state security.

The remainder of this article details our research methods for public health apps and then explores relevant apps in our case studies of China, Australia and NZ. This analytical work allows for a critical inquiry into the dynamics of platform operation as they vary across regions and process sensitive health information and geolocational data. The discussion that follows reflects on these approaches and considers the epistemological assumptions of these and other responses of digital public health surveillance.

## Research design

Our research design considers how best to enquire about interface and politics within 21st-century pandemic governance. Tarleton Gillespie’s (2010) ‘The Politics of “Platforms”’ argues that platforms can be understood as socio-technical assemblages and complex institutions which involve multiple public and private actors. We are also informed by the work of Keating and Cambrosio (2003) as their epistemology of health technology focuses on the way that platforms are infrastructural systems that come to conclusions about complex scenarios, and pass those conclusions as objects along to other platforms to administer interventions. In this sense, we approach COVID tracing applications as important parts of health infrastructure that determine the likelihood of illness, before passing the responsibility of action on to other platforms – such as the health or legal system. At the same time, there is a rhetorical and user experience level to these tracing applications that inform their governance capacities over citizens. As such, our research activities are informed by the above epistemologies, but also by methodologies, such as the walkthrough (Light et al., 2018), media go-along (Jørgensen, 2016) and scrollback methodologies (Møller and Robards, 2019), which treat the application interface seriously. While we are informed by these methods, we are addressing a different type of system, which does not share the generally perceived aspects of sociality and interaction found in social media platforms. Instead, we treat each app’s semiotic interface and links to public policy as a window onto how the governing health platform operates.

We organise the research focus around three primary attributes of each app, guided by app-based walkthrough methods (Light et al., 2018). First is the ‘vision’ that establishes a discursive and semiotic account of how the app frames people at different points (as users, citizens, disease vectors or otherwise) and how this frames the user base and its connected communities. Second, the ‘operating model’ focuses on the political–economic structure of the app, investigating how it extracts data from user input or passive surveillance systems and leverages these results to hierarchies of (political/economic) power. The third is the ‘governance’ of the platform, where researchers investigate how the platform controls, promotes or limits particular types of engagement both in-app and beyond via the technicity of the app or through post-factum interventions with power (policies, infrastructures, kinetics). These attributes have similar manifestations in Gillespie’s (2010) work when framed as ‘users, advertisers and clients’, ‘policy’ and ‘edge’, but we retain the walkthrough language for clarity of analysis. Reporting on these groupings of data enables analysis through frames of symbolism, technical epistemologies and material impacts. Our approach thus also draws out the discursive and semiotic encodings about how apps describe their own ability to protect or prevent disease; we study the technical functions in relation to the particular epistemology of disease that is implied; and finally we examine how the app is put into practice with actual healthcare interventions – or lack thereof.

The choice of Australia, China and NZ is based on our own positions as researchers linked to these countries through citizenship and/or geography. Our research commenced in April and lasted to the end of September 2020. First, we established each platform’s environment of expected use by drawing upon government press releases and popular media to examine the platform’s vision, operating model and governance. Second, we conducted a technical analysis based on each app’s architecture separately, taking screenshots and fieldnotes as we stepped through the registration process on the platform; examined user interface arrangements; analysed textual, symbolic content and tone; and engaged in everyday use. We aimed at documenting the dominant features, the possibilities afforded by their functions and the socio-political implications of app operations for managing the pandemic. While the authors each have cultural, discursive and/or familial ties to the nations studied, we do not claim ethnographic knowledge of being on the ground, with apps installed outside of Australia. We relied on published commentary and private messaging to author(s) about the functionality of working and living with these COVID tracing apps in China and NZ and our own experiences in Australia. Our approach reflects the material entanglements that the apps produce at a level abstracted to public health. This means it can prioritise the researcher’s narrative of interpretation while undermining the diverse user-centric approach to the platform (Dieter et al., 2019); the abstraction to public health imaginary presents a limit but also a unique opportunity to situate the work in the midst of a global pandemic to conceptualise public app use. Our analysis remains interested in considering larger socio-technological questions on *how* and *why* these apps define their purpose, user base and scenario of use, underlying political and economic interests, and management and regulation of activity, instead of vernacular cultural practices (Grimes, 2015) or departures from intended use.

Our approach thus builds from methods designed to intervene in managing social relations in digital spaces to trace the development of interventions designed through specific apps in the context of disease-management applications. We seek to explore the vision, governance and operating model of COVID-19-related applications and like the media go-along method (Jørgensen, 2016), we must start in *media res*. Because the apps we study are limited in terms of user feedback, the discussion below departs from the socio-political architecture of the COVID tracing apps, but rather investigated their function in terms of influence on healthcare governmentality.

## China’s Health Code on WeChat and Alipay

In China on 20 January 2020, the Spring Festival holiday was interrupted by a nationwide lockdown that sought to intercept the flow of a massive number of people that were likely to spread a newly discovered disease. During this time, at least 760 million residents were confined within their households (Hessler, 2020). Nationwide restrictions were imposed in different cities and provinces according to the COVID-19 emergency levels, the evaluation of which was based on the number of deaths and confirmed cases, as well as the extent of intra- and inter-province migration flows before the national holiday (Caixin, 2020). The principle here was that cities and provinces with high numbers of infections and deaths or with significant population movement were classified as high-risk areas. Wuhan, Heilongjiang, Shanghai, Beijing and others gained particular attention from provincial authorities and, subsequently, received China’s highest level of public health emergency response. The ‘Level 1’ designation meant that these cities were to be administered by the national central government (Yin and Ning, 2020), losing their provincial autonomy.

China’s economy was paralysed for months during the national lockdown; governments at national and municipal levels set about preparing for the economic recovery from the pandemic. The solution was ‘Health Code’, effectively a national-level digital equivalent to a *Carte Jaune* travel inoculation passport, specific only to COVID-19, and only in terms of whether the possessor was likely to be infected with the disease. Health Code was derived from a function on Alibaba’s DingTalk, an app that involves employers monitoring their workers’ health records. In early February 2020, Hangzhou municipal government in Zhejiang extended the Health Code function to its citizens in collaboration with Alibaba, integrating the Zhejiang provincial Health Code into Alipay. Concurrently, the municipal government in Shenzhen, where Tencent is based, instead formed a partnership with WeChat seeking a technological solution for economic recovery from COVID-19, leading to a second Health Code integrated into WeChat’s social media systems, rather than Alipay’s e-commerce systems.

On both Alipay and WeChat, Health Code operates in the form of mini-program integrated into existing app services. Mini-programs are common across these platforms and are known for their small file size (10 MB), low development cost and low traffic requirement (Graziani, 2019). Unlike other mini-programs, Health Code programs are automatically added to users’ WeChat and Alipay services, leaving users incapable of deactivating the function, without abandoning the service. This mandatory aspect contributes to the high saturation of the programs across user accounts, contrasting sharply with commercial or institutional mini-programs that are subject to optional subscription or installation. While initially named ‘Anti-Virus Code’ (bingdua 病毒码) on WeChat in mid-February, the program was later relabelled as ‘Health Code’ (jiankangma 健康码), a term generating much positivity, as it was rolled out within Zhejiang and Guangdong provinces where the two tech firms are located. On 29 February, Health Code was officially launched nationwide by China’s State Council as a technology for controlling the post-pandemic population movement. The widespread use of WeChat and Alipay supports the Chinese government’s aim to instal Health Code across some 2.1 billion registered accounts, including 1.1 billion and 1 billion registered accounts, respectively, on WeChat (CIW Team, 2019) and Alipay (Xinhuanet, 2019), calculated in the first quarter of 2019. Thus, Health Code systems cover as much of the population of China as possible. The aim here is for the Health Code to sufficiently cover travellers within China as well, such that the country’s economy will effectively return to the old norm where mobility and economic activities are possible.

**Figure 1.**
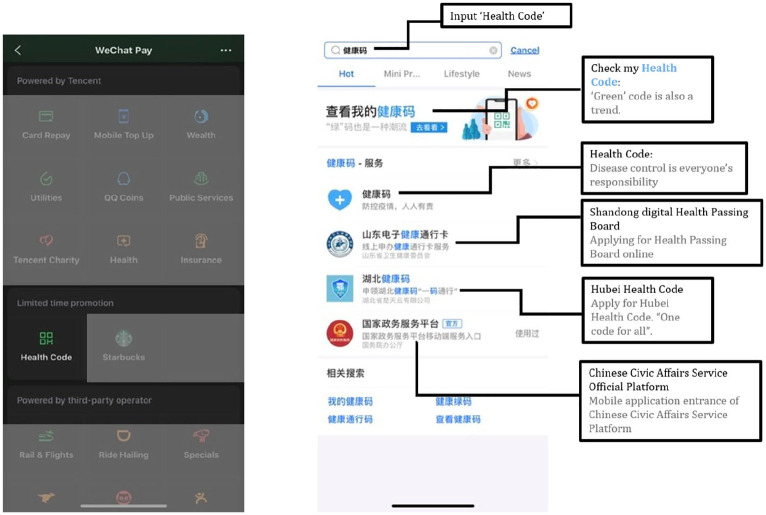
Screenshots of Health Code on WeChat (left) and Alipay (right).

The implementation of the Health Code service on both WeChat and Alipay (Figure 1) involves a tracking feature. This feature is used differently in the different regions around China depending on the overlapping local, provincial and national implementations of movement controls: the use of Health Code services will depend on both national prerogatives and provincial implementations (Mozur et al., 2020; Xinhuanet, 2020). At the national level, the method for determining a person’s health status within the categories of ‘mandatory quarantine’ (red code), ‘self-isolation’ (yellow code) or ‘free movement’ (green code) within the Health Code mini-program would appear to be the same, yet the way that this is managed and described differs from province to province. Exploring different geolocations through the Health Code service, we found that in regions under a ‘Level 1’ emergency designation, mobility is highly restricted, and the Health Code service is described as ‘powered by the regional government’. Those in regions with lower emergency ratings have proportionally greater degrees of intra- and inter-provincial travel, and the Health Code service is correspondingly described as ‘powered by the national government’. This simultaneously refers to not only devolution of crisis management to the provincial powers but also devolution of responsibility from the central administration to the provinces.

The differences in the Health Code service remain diverse within the provinces, sectors and municipalities across China (multiple government initiatives at different levels have recently created local versions of Health Codes; see Law, 2020), as well as being complicated by emerging Health Codes provided by specific government bodies (including China’s Health Commission, Transportation, Immigration, phone carriers and Civil Aviation Administration; see Xinhuanet, 2020) that operate at different organisational levels. Furthermore, the method of determining a user’s risk category is inconsistent across the various platforms, leaving a user identified as ‘at risk’ on one platform, while having ‘free movement’ on another (Davidson, 2020). The organisational structure of the Health Code system and its use is complex and lacks standardisation across China and represents a significant limitation to the ability to study the individual experiences of Health Code service via the research method, given the great diversity of experiences and implementations.

The Health Code system powered by WeChat and Alipay is constrained by its domestic application and the inability of mutual recognition in the cross-provincial context. In April, China’s Ministry of Industry and Information, collaborating with three state-owned telecommunication companies China Telecom, China Mobile and China Unicom, and a research institute The China Academy of Information Communication Technology (CAICT), launched Communication Travelling Code (tongxinxingchengma 通信行程码) mini-program on WeChat for international travellers to China. Native apps renamed as Communication Big Data Travelling Code (tongxindashujuxingchengka 通信大数据行程卡) were simultaneously launched on iOS and Android systems. These technologies remain inaccessible to users who are geographically identified outside mainland China. The later update of Communication Big Data Travelling Code in May intersected a Bluetooth approach to ‘alert’ users of confirmed and suspected infections nearby (CAICT, 2020) steering China’s Health Code system towards a similar operating system to Australia’s COVIDSafe.

## Australia’s COVIDSafe via Appstore

On 19 January 2020, a traveller from Wuhan arrived in Melbourne, and soon thereafter presented relevant symptoms to public health authorities. Ten days later, Australian scientists using that sample were the first outside of China to grow, sequence and share a severe acute respiratory syndrome coronavirus 2 (SARS-CoV-2) genome (Caly et al., 2020). This early scientific surveillance moved in parallel with the policy that stopped direct flights from China by the 1st of February, and then other countries through March as global infection rates rose. In the next 60 days, Australian infection rates from returning travellers and community transmission followed then-common epidemiological doubling rates (between 3 and 5 days) seen in the first wave of pandemic, but stalled out after 60+ days; the country was largely self-isolating before state and national governments moved to restrict gatherings and induce fines for leaving one’s home by the end of March (Seccombe, 2020). From this context, on 14 April, the federal government announced the tracing app COVIDSafe that built on Singapore’s TraceTogether app’s apparent success (Remeikis, 2020) and the underlying OpenTrace/BlueTrace Bluetooth protocol (OpenTrace, 2020). The first version of COVIDSafe was available for download in Australian App stores for iOS and Android devices 12 days later, with later updates in regional app stores to open functionality to international people in Australia. It should also be noted that the government’s repeated stated vision of COVIDSafe was focused as much on enabling economic activities insofar it is related to public health (Goggin, 2020), which mirrors the Morrison government’s more general messaging on threats to Australia from the pandemic.

**Figure 2.**
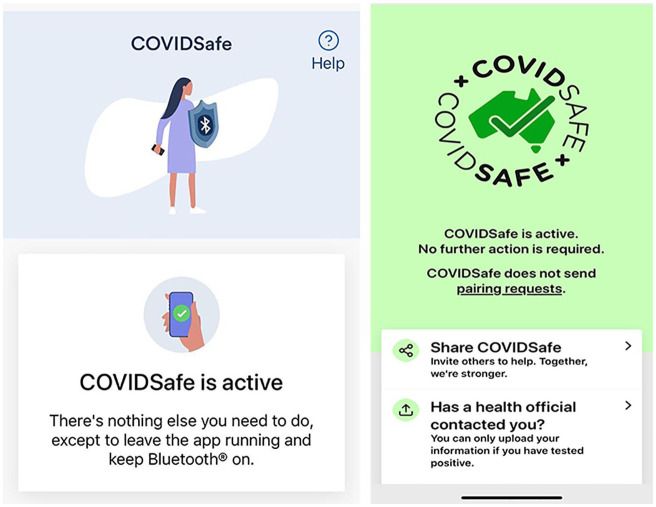
Screenshots of the version 1.0 launch screen (left) and the updated version 1.5 (right).

Initial activation of the app carries the user through a narrative via 10 click-through screens. After the first brand image launch screen, the next page explains public health utility and an action button that according to its label, affords, ‘I want to help’. The subsequent pages whisk through technical, privacy, policy, registration consent and demographic data, with an authenticity check via mobile number SMS and two-system interactions to enable Bluetooth and notification permissions. Users then receive a successful registration message and are alerted that the app has become ‘active’ with links to further informational resources scrollable below (Figure 2).

When operating, the app functions as a background system without notable usability, aside from routinised alerts at 9:00 a.m. (AEST) specifying the need to maintain active Bluetooth capacity by keeping the app ‘open’ in the background. The alert is framed to an active subject with the language ‘We need you!’. Upon activation, the first versions of the app showed a gendered female avatar in a knee-length smock (notable for its lack of pants/leggings and distinct white sneakers) holding a large metal heater shield in one hand and mobile phone in the other. The Bluetooth icon sigil on the shield visually symbolised the protection of the tracing app and the subject being protected. Later versions replaced the shield and avatar with a ‘check-marked’ map of Australia, protected by an encircling animated message: ‘**COVID**SAFE x COVID**SAFE** x’ (Figure 2). The abstraction from an individualistic gendered image to the national border protection aligns with Western ideology and Australia’s specificity to sovereignty and protection of its borders. It also plays to recent anticipatory governance concerns around efficacy in pandemics (see French and Monohan, 2020). These images function in the Australian context as a discursive device that emphasises among other regimes, ongoing colonial power and a filter for specific migrant bodies within Australian territory (Chambers, 2015; Wilson and Weber, 2008). Here, the Australian nation is gendered and protected with the tracing technology or represented as a self-isolated state without coronavirus contagion.

The discursive cues of active citizens and bordered subjects are matched in many of the technical functions of the app itself. Unlike China’s Health Code, COVIDSafe eschews connections with individual citizen information by producing randomised identifiers that are refreshed every 2 hours. These identifiers are broadcast between devices that record (the strength of specific) Bluetooth signals from each other and are stored on devices for 21 days. These local identifier data are only actioned under the condition of a user (a) being contacted by a health official to communicate a positive COVID test outcome and send a unique activation pin via SMS and (b) the user then being able to upload locally stored IDs as encrypted data to a government server, run by Amazon Web Services (Australian Government Department of Health, 2020). Entering the received PIN functionally delivers context-aware active and informed consent of the app’s intended public health use.

Reported functional success of Bluetooth identification between devices varies across iOS/Android; it is rated to work 50% to 80% of the time by Australia’s Digital Transformation Agency (as of May 2020 and the four versions released to improve Bluetooth functionality). For context, as of submission (October 2020), there are more than 7 million instals and two waves of SARS-CoV-2 that had 7000 and 20,000 COVID cases, respectively, but only 14 tracing successes through the app. The precise scale of app deployment remains obscured by the federal government claiming ‘privacy’ concerns (Taylor, 2020) and the efficiency of the app remains unproven through a single case. Interestingly, all of the 14 traces were from one state in Australia, which did not have the highest infection rates – the Victorian State Government tracing resources eschewed its use due to perceived lack of utility, even during their outbreak which peaked at 500 to 700 new cases a day (Swan and Grifith, 2020).

Governance around COVIDSafe has shown surprising contextual integrity for user data and openness to implementing feedback, considering the Australian government’s previous surveillance compulsion (Mann and Daly, 2019). Government spokespersons have repeatedly acknowledged the voluntary nature of downloading and using the app, with the Health Minister offering that the alternative would have ‘breached the partnership’ with the public. The source code for the app (but not central repository server) was shared on GitHub after vociferous feedback from national privacy experts and non-governmental organisations (NGOs), who ran citizen audits on the app and code as they became available (Glenn, 2020). Novel to Australian law regarding retention of citizen data is the illegality of sharing any COVIDSafe data with any agency or using it for any purpose other than the specified tracing; data will be deleted once tracing activities are stopped. This is surprising considering previous Australian regimes of data retention and utilisation are fraught with controversy, ineptitude or outright illegality (Mann and Daly, 2019; Wilson, 2020; Wolf, 2020). From this context, deployment of a government COVIDSafe app required a measured approach where any potential post-factum power intervention over its subjects was undesirable.

## NZ APP

A different approach still is taken by the NZ COVID Tracer app (Ministry of Health, 2020), which does not make use of any sort of near-field communications system. It uses neither Bluetooth nor near field communication (NFC) nor commercial application programming interfaces (APIs). The NZ COVID Tracer app instead works through QR codes generated for businesses and organisations when they register with the app. The official Ministry of Health QR codes are placed at the premises for people to scan when they arrive and add the location to their ‘digital diary’ of movements, providing a personal historical trajectory of contact points. Yet unlike Australia/China, the process is neither automated nor mandatory, and while some users apparently have issues with scanning (see reviews at iOS store and Google Play), the service operates to both alert locations and users of any infection risks. In the event of any kind of infection, the NZ COVID Tracer app allows users to securely share their check-in history with a registered ‘Contact Tracer’. The disease epistemology of the NZ COVID Tracer app is different from both the Chinese and Australian context. The Australian COVID Tracer app context is largely borne out as an idea of transmission as a deracinated network of individuals. The Health Codes in China have varied approaches to individual health, but priority is given to people attempting to move between distinct zones. The NZ COVID Tracer is something of a synthesis of these two positions, adopting a concern for spaces first and individuals second. This focus on particular destinations as disease risks as nodes within a network involves a socially sorted geographic mapping (Graham, 2005) of parts of NZ as being network points. This epistemology is distinct from both the Health Code service and the Australian COVIDSafe because it includes small, bounded sites into the logic of disease mapping, thus framing surfaces as well as individuals as sites of potential infection.

**Figure 3.**
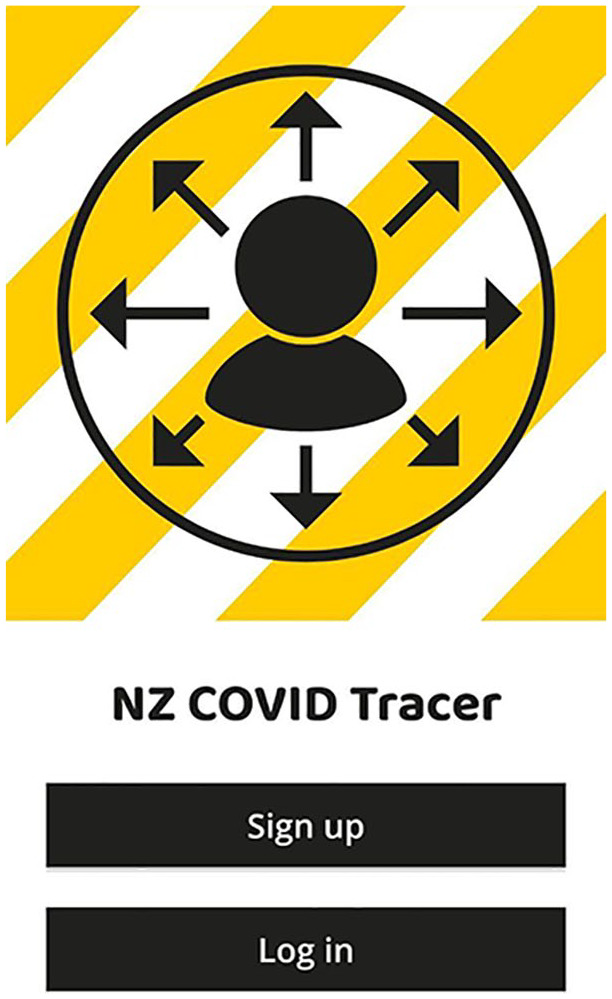
Screenshot of the launch page of NZ COVID Tracer.

The discursive materials that surround the NZ COVID Tracer app lack specific medical symbols. There are no symbols representing a shield, no image of a healthcare symbol or even an idea of inoculation. The strongest didactic language is limited to ‘Protect yourself, your whānau, and your community’ (whānau meaning family in Te Reo Māori), located on the same tab as the button for scanning QR codes. This implies a protective model but particularly emphasises the relationship between the user and their immediate social groups, and highlighting that health in this context is not solely an individual thing. This is reinforced in some of the symbolic imagery (Figure 3) that is associated with the application itself, the image of the individual in a bubble surrounded by radiant arrows, with a yellow/white striped caution background. This symbol connects to the predominant health discussions in NZ about managing personal health ‘bubbles’. The Ministry of Health’s (2017) New Zealand Influenza Pandemic Plan (2002/2017) notes that a key mechanism for managing a pandemic in NZ would involve the development of a social distancing framework (pp.79-91) that emphasises the reduction of travel and selective community contact. In the current climate, this has been communicated by the NZ Government as a logic of ‘bubbles’. Social distancing is not a complete social isolation of everyone, but instead encourages the people in the community to establish their own enclaves of sociality. Groups are encouraged to maintain their bubbles to reduce their chance of infection and to reduce the overall rate of infection. This symbol, of the individual in the bubble, represents this concept and is the main image that users encounter when logging in to the NZ COVID Tracer app.

## Discussion

Each of the applications within China, Australia and NZ imply a particular role for digital media within assemblages of medical governance. This occurs alongside epistemologies of media and disease, and theories of risk, individuation, community and nation. The apps and their implementation are not solely technical systems but are summoned into public consciousness and the individualisation of health responsibility. These contexts interplay with technical solutions to develop new semiotic and discursive expectations of these services being presented as specific solutions. Publics form around – or against – these modalities of health governing.

Pandemic surveillance technologies engage in the ways that humans self-constitute their practices as health-conscious citizens. The systems are not about health but coded and coding health through these tracing technologies where citizens are de-subjectified when being represented by health codes or data points. Free installation, easy registration and login, and background operation without draining phone battery underline both technical and discursive aspects of the investigated COVID tracking technologies. They collectively put values of efficiency, cost-effectiveness and user speculation at the fore over other legitimate concerns such as data storage, protection of health record, social justice, non-discrimination, equity, digital literacy and universal accessibility to digital devices. The emphasis on ‘scientificity’ with the extensive use of technical terms like ‘big data’, the sole involvement of technical objects and immediate contacts with health workers further veils the back-end operationalisation of COVID tracing apps to the public and detaches government bodies and tech corporates from accountability mechanisms.

Public health is not individual health; it is a novel subject for regulation during the pandemic. Yet, a neoliberal discursive framework of health consciousness plays a role in individualised responsibility in Australia and NZ through an encouraging framing of language that intends to incorporate many citizens into the surveillance assemblage. Such governmentality within the context of COVID-19 crisis is neither deemed as being overtly coercive nor forceful but rather as operating on autonomous individuals willingly regulating themselves in the best interest of the state (Lupton, 1999) despite the misalignment of public values and private interest. In contrast, the positivity generated from China’s mandatory Health Code system manifests the expansive state surveillance as the new normativity. The function of this norm, operated differently from the law, is associated with appositive technique of intervention and transformation to this nationwide normalisation campaign (for an extensive accounting of the concept of normativity, see Pasquinelli, 2015).

The goal envisioned in the pandemic surveillance technologies investigated in this article is economic recovery contingent on lifted restrictions and population movement at various scales. This narrative draws a socio-technical correlation between returning to normal economic activity and the necessity of pervasive surveillance technologies. These correlations become increasingly visible in later waves of increasing unemployment and COVID-19 infections that match the futility of the massive digital surveillance over humans for a pathogen with a 14-day life cycle. At the time of writing (September 2020), cities in China, Australia and NZ with more scalable and frequent population mobility have been experiencing the resurgent outbreaks despite the strict biological governance over their citizens implemented through the pandemic surveillance networks. It appears that there exists a delicate balance between this form of public health ‘protection’ and the rationale of economic recovery. We see a line to the argument that these surveillance technologies are purported to maintain the totality of the nation-state for harnessing their instrumentarian power (Zuboff, 2019: 686–691). The argument is framed such that a lack of visibility and accountability of citizens equates to depressed economic circumstance. A comparison with post-9/11 surveillance can be instructive in this context when claiming security has been practised through the ongoing war on terror while excessively compromising the privacy of ‘others’. Yet in 2020, surveillance is freedom, with the most egregious abuses of power during the pandemic so far coming not from states that maintained strict control over the mobility of their citizens (cf. French and Monahan, 2020) but those that have been intentionally denying the benefits of mass testing, contact tracing and more radical surveillance measures as seen in the United States and Brazil.

Users of COVID tracing technologies are turned into sensors of each other through locational-aware functions. Their surveillance trade-offs and indeed an epistemology of NPIs through national mobile phone apps are not the only models of digital pandemic surveillance that exist. A variety of surveillance technologies are being experimented or implemented across the world with the aim of controlling the virus (Budd et al., 2020). Despite their limited utility during the lockdown, they could prove more appealing when movement restrictions are lifted, yet also expose more privacy-related concerns. For example, Singapore has revisited its reliance on app-based trackers and their related privacy and phone ownership concerns to institute hardware-based ‘dumb’ Bluetooth tags for the public that do not use Internet connections or GPS chips. These wearable tracing devices are handed over to healthcare professionals for their Bluetooth proximity data (O’Donnell, 2020). This physical barrier to sharing anonymised surveillance information on fellow citizens presents a different logic than assumptions common in critiques of surveillance capitalism (Zuboff, 2019), which focuses on the parasitic existence of capitalist systems with its reliance on data collection. The physicality of this specific digital tracing device substitutes a disconnection from a networked ‘corona’ of the Internet-connected individuals, and instead only makes visible post hoc physicality networks that create new subjects for public health concern. Here, the individualisation of public health is transferred from the relatively easy active consent of installing a virtual app, to an increased physical presence but one that has less networked privacy concerns. While this approach removes the Internet connection to reduce privacy concerns, another approach subtly distances the state from public health data instead.

Platforms of public health surveillance for COVID-19 tracing are not limited to the national contexts, which indeed shows limits of public health as relayed by state structures. In May, Apple and Google proactively engaged in helping pandemic surveillance through an extraordinary joint effort to create a ‘Privacy-Preserving Contact Tracing Project’ (Google, 2020). This provided an API to dissociate individual implementation of tracing apps from individualised public health relations vis-à-vis the state. Instead of an app, the system uses a set of platform-independent standardised APIs integrated into iOS and Android operating systems for interoperability with national contact tracing services (via their apps). Unlike our other examples, the relationship between the APIs’ function and any materiality of disease transmission is articulated in a more limited and precise way. As the joint announcement notes, the problem to be solved less than a full-spectrum accounting of disease transmission and protection of economic interests, and instead focuses on technical issues related to ‘Exposure Notification’ through Bluetooth, cryptographic and API frameworks (Apple, 2020). We note these limits to digital tracing utility are mirrored in language of the lead developer of Singapore’s BlueTrace technology, which was implemented in the Australian tracing app. He states such methods are not a panacea and ‘any attempt to believe otherwise, is an exercise in hubris, and technology triumphalism’ (Bay, 2020).

Google and Apple’s surveillance *platform* became more visible, when in mid-2020 they dropped the requirement for an app to be installed at all for tracing to work. It was now possible for ‘Health Authorities [to] inform users of potential exposure to COVID-19 without a dedicated Exposure Notification app’. The tracking capacity was seamlessly included into the Operating System for potential health tracing needs; Apple lets users opt-in. As of October 2020, over 40 government apps now utilise the Apple–Google API (Siddiqui and Rahman, 2020) in a pattern of growth that mirrors the global market positions of these firms’ incumbent platforms. How health surveillance evolves living with COVID-19, and the role of the state or lack thereof, is a critical question for platform studies and public health sociology to embark on.

Nor, at this stage, it is likely that pandemic surveillance technologies will not be easily abandoned by some states. For instance, future extensive use of Health Code has been proposed by some municipal governments in China; this proposal has introduced public contestations regarding the government access to health data while expressing their concerns about the further combination use of Health Code and the social credit system that can ‘quarantine’ certain groups of people by diminishing their equal access to public facilities or services. This article serves as a point of departure as we believe that the novel tracking technology discussed will inevitably shape future discussions of surveillance during and after the COVID-19 pandemic.

To describe these shifts in surveillance, we have proposed the ‘corona’ of pandemic surveillance to indicate the way that individual broadcast a halo of information about themselves, that has become a somewhat socially permitted government intervention in the name of healthcare. The corona of the virus is mimicked by the corona of data from each individual, shedding not just viral loads but also data packages. This sets capacities and constraints for the good life into the future – ostensibly in interest of public health and continuing economic recovery. Now different strata within the states, governments and transnational tech corporations have developed pandemic surveillance technologies. Even in itself, the corona of data is infectious, presenting a model of surveillance that could reterritorialize social expectations around data use, and providing a sufficient rationale of health and safety.

## Conclusion

This article has profiled tracing systems instituted for 2020’s novel coronavirus pandemic in terms of their novel surveillance goals on public health grounds via platform critique. We specified the disparities in approaches to app-based contact tracing employed in China, Australia and NZ, through consideration of the varied actors and structures on (and with) citizens involved. Our comparative analysis of these apps and review of other appendages of digital and surveillant NPIs for the pandemic offer crucial frames to consider unfolding questions of citizenship, privacy and government for a changing world. The digital materiality of public health tracing refracts surveillance and citizenship through a set of novel ‘coronas’ of surveillance for governing surfaces of the public through what emanates from their private bodies. Configurations of citizenship, privacy and government in the world changed by pandemic requirements are evolving, with both new actors and goals being explored and experimented with in terms that signify concepts of public health. Each of the differences between platforms and systems implementation and various views to disease epistemology complicate the space and goals, while redefining what humans and their relevant coronal-auras are expected to do in larger systems of democratic and economic control.

## Notes

1. Spanish for crown, the etymology of corona is striking in that sciences of the *body* see crown like appendages, while sciences of *radiation* see coronas as light or charged particles; health surveillance here affects both the body’s appendages (phone) and its radiating data.
